# Esketamine Combined with Propofol TCI versus Propofol TCI for Deep Sedation during Endobronchial Ultrasound-Guided Transbronchial Needle Aspiration: A Prospective, Randomized, and Controlled Trial

**DOI:** 10.1155/2023/1155126

**Published:** 2023-12-11

**Authors:** Sichen Cui, Peiying Huang, Zhanxiong Wei, Ting Guo, Aiyan Zhang, Lining Huang

**Affiliations:** ^1^Department of Anesthesiology, The Second Hospital of Hebei Medical University, Shijiazhuang 050000, China; ^2^Department of Pneumology, The Second Hospital of Hebei Medical University, Shijiazhuang 050000, China

## Abstract

**Background:**

Endobronchial ultrasound-guided transbronchial needle aspiration (EBUS-TBNA) is an invasive procedure that required deep sedation to suppress coughing and body movements. Deep sedation, on the other hand, has been shown to cause respiratory and circulatory depression, especially when the airway is shared with the endoscopist. Esketamine is a novel sedative and analgesic with little respiratory inhibition that appears to be an appropriate adjuvant in propofol sedation for EBUS-TBNA. We compared the efficacy and safety of esketamine combined with propofol target-controlled infusion (TCI) and propofol TCI for deep sedation in EBUS-TBNA.

**Methods:**

The study included 135 patients with ASA II-III undergoing EBUS-TBNA. They were randomly divided into two groups (group E and group P). Both groups received midazolam (0.01–0.03 mg/kg) and oxycodone (0.07–0.08 mg/kg). Then, patients in group E received 0.3 mg/kg esketamine, propofol TCI, and 0.2 mg·kg^−1^·h^−1^ esketamine for sedative maintenance. Patients in group P received only propofol TCI. The primary outcome was the dose of 1% lidocaine administrated by the endoscopist and the times of lidocaine sprays. Secondary outcome indicators were cough score, propofol dosage, patient satisfaction, endoscopist satisfaction, the incidence of sedation-related adverse effects and side effects, and recovery time.

**Results:**

Patients in group E were given significantly less lidocaine (4.36 ml/h (2.67–6.00) vs 6.00 ml/h (4.36–7.20), *P* < 0.001) and less spraying frequency (2.18 times/h (1.33–3.00) vs 3.00 times/h (2.18–3.60), *P* < 0.001) than group P. There was a statistically significant difference in cough score between the two groups (group E 2 (0–4) vs group P 3 (2–4), *P*=0.03). Also, mean arterial pressure (MAP) was higher in group E in the 30^th^ min (T5, 84.10 ± 12.91 mmHg versus 79.04 ± 10.01 mmHg, *P*=0.012) and 40^th^ min (T6, 87.72 ± 15.55 mmHg versus 82.14 ± 10.51 mmHg, *P*=0.026). There were no significant differences between the two groups in terms of sedation-related adverse events and side effects, recovery time, endoscopist satisfaction, and patient satisfaction.

**Conclusions:**

In patients with ASA II-III, esketamine as an adjuvant in combination with propofol TCI deep sedation for EBUS-TBNA can improve the sedation effect, reduce coughing reaction during the procedure, and obtain more stable blood pressure. No reduction in the occurrence of sedation-related side effects was observed. This trial is registered with ChiCTR2200061124.

## 1. Introduction

Endobronchial ultrasound-guided transbronchial needle aspiration (EBUS-TBNA) is a minimally invasive procedure for taking needle biopsies of lung and peripulmonary tissue utilizing real-time ultrasound guidance. It is now used more commonly in the diagnosis and staging of mediastinal lymphadenopathy due to its safety and higher diagnostic rate compared to bronchoscopic transbronchial biopsy [1, 2]. Since the process is generally lengthy, unpleasant, and extremely irritating to the airway, it is preferably conducted under sedation [3, 4]. Previous research has indicated that deep sedation increases diagnostic yield and the amount of lymph nodes examined compared to moderate sedation [5, 6].

The most effective, safe, and satisfying sedation regimen for EBUS-TBNA remains unknown at this time. Midazolam, propofol, opioids, and local anesthetic agents such as lidocaine are commonly used during EBUS-TBNA and are generally given in combination to achieve better sedative efficacy [7]. However, the combination of these agents can lead to cardiovascular and respiratory depression, especially when anesthesiologists share the airway with endoscopists [8–10].

Esketamine, as the pure s-enantiomer of ketamine, has been widely used in clinical analgesia and anesthesia [11–13]. It produces sedation and analgesia by blocking N-methyl-D-aspartate receptor and has both cardiac excitatory [14] and bronchodilatory qualities, which can counteract the cardiopulmonary depression of propofol. We anticipated that using esketamine as an adjuvant during EBUS-TBNA would result in more steady sedative efficacy while reducing the dosage of other sedative drugs and sedation adverse effects.

This study was designed to evaluate the clinical efficacy and safety of esketamine combined with propofol target-controlled infusion (TCI) during deep sedation for EBUS-TBNA. The dose and frequency of lidocaine sprayed by endoscopists during the procedure was chosen as a substitute parameter for sedative effectiveness. Secondary outcomes included other effectiveness parameters (cough score, propofol dose, and patient and endoscopist satisfaction), safety parameters (respiratory and hemodynamic effects), recovery time, and side effects (vomiting and psychotomimetic side effects).

## 2. Methods

This article adheres to the applicable CONSORT guidelines. The study is a prospective, randomized, and controlled trial performed in the Hospital of Hebei Medical University from June 2022 to January 2023. The protocol for the study was approved by the Institutional Ethics Committee of the Second Hospital of Hebei Medical University (2022-R278), and written informed consent was obtained from all subjects participating in the trial. The trial was registered prior to patient enrollment at the Chinese Clinical Trial Registry (ChiCTR2200061124, principal investigator: Lining Huang; link to trial registry: https://www.chictr.org.cn/edit.aspx?pid=171923&htm=4; date of registration: June 15, 2022). The complete data used to support the findings of this study are available from the corresponding author upon request.

The study included consenting patients aged 18 to 89 who underwent EBUS-TBNA and were classified by the American Society of Anesthesiologists (ASA) as II-III. Exclusion criteria included body mass index >28 kg/m^2^, difficult airway, history of cardiovascular or cerebrovascular adverse events in the previous six months (including poorly managed hypertension, severe arrhythmia, myocardial infarction, stroke, or TIA symptom onset), cognitive dysfunction or mental illness, complicated liver and kidney disease, hypoxemia (resting oxygen saturation <90%), and inability to give informed consent.

**Figure 1:**
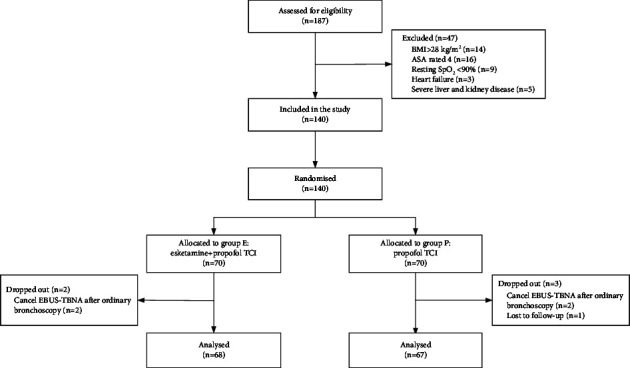
Consort flow diagram.

A total of 187 patients were evaluated for eligibility, and 140 were enrolled following signing the informed permission form. Patients were randomly allocated to one of the two groups (randomly assigned 1 : 1): esketamine-propofol TCI group (group E, *n* = 70) or propofol TCI group (group P, *n* = 70) (Figure 1). Randomization was done using a computer-generated random sequence sealed in an envelope. Anesthesiologists were not blinded, but all patients, endoscopists, and endoscopic nurses were blinded to the grouping.

All patients fasted for 8 hours before EBUS-TBNA. In the operating room, patients took a supine position and received oxygen (4 L/min) via nasal cannula. Blood pressure, oxygen saturation (SpO_2_), and electrocardiogram were monitored. After the placement of an intravenous line, 0.5 mg of penehyclidine hydrochloride (Jinzhou Avanc Pharmaceutical Co., Ltd., Jinzhou, Liaoning, China) was injected. Five minutes before sedation, a 2% lidocaine (Tianjin Jinyao Pharmaceutical Co., Ltd., Tianjin, China) and 1% tetracaine (Chengdu Tiantaimount Pharmaceutical Co., Ltd., Chengdu, Sichuan, China) mixture was sprayed three times on the oropharynx via spray pot, and a dose of 3 ml on the glottis via thyrocricocentesis. Midazolam (Jiangsu Nhwa Pharmaceutical Co., Ltd., Xuzhou, China) (0.01–0.03 mg/kg) and oxycodone (NAPP Pharmaceuticals Limited, Cambridge, CB4 0 GW, U.K.) (0.07–0.08 mg/kg) were given to all patients, and three minutes later the propofol TCI was started. We used the target-controlled syringe pump (CP-700TCI) preprogrammed for propofol (Xi'an Libang Pharmaceutical Co., Ltd., Xi'an, China) infusion in the Marsh model.

In group E, the initial propofol plasma target concentration was set at 1.4 *μ*g/ml. A bolus of esketamine (Jiangsu Hengrui Pharmaceuticals Co., Ltd., Lianyungang, Jiangsu, China) 0.3 mg/kg was given intravenously as propofol began to infuse and pumped at a constant rate of 0.2 mg·kg^−1^·h^−1^ during the procedure. In group P, the initial target concentration was set at 2.0 *μ*g/ml. The sedation level was accessed by the Modified Observer's Alertness/Sedation scale (MOAA/S). Lower scores in the table represent deeper sedation. A score of 5 indicates a sensitive response to a name call in normal tone, a score of 1 indicates only a response to a painful stimulus (squeezing at the trapezius site), and a score of 0 indicates no response to a painful stimulus. The endoscopic procedure began when the initial target concentration was reached or the MOAA/S score was 0. During the procedure, MOAA/S was accessed every 5 min, maintaining a score of 0-1 by adjusting the target concentration of propofol. When oxygen desaturation occurred as a result of glossoptosis, oxygen delivery was increased to 10 L/min, chin raise, jaw thrust, and placement of a nasopharyngeal airway tube were given if necessary. Corresponding therapeutic applications were also given to other sedation-related adverse events. The pump injection was stopped 5 minutes before the end of the procedure. After the procedure, all patients received flumazenil 0.5 mg and were then sent back to the ward when the modified Aldrete scores (MAS) reached 9.

The primary outcome was the dose and times of lidocaine given by the endoscopist. The endoscopist had the right to spray 1% lidocaine through the bronchoscope any time it was necessary according to the coughing response of patients. We set a limit of 2 ml per spray and a maximum dose of 7 mg/kg of lidocaine.

The secondary outcomes were the incidence rate of hypoxemia (SpO_2_ < 90% for more than 20 s), hypotension (drop in MAP < 20%), hypertension (increase in MAP > 20%), tachycardia (HR > 120 bpm), bradycardia (HR < 50 bpm), malignant arrhythmia, esketamine-related psychiatric symptoms, and nausea and vomiting. Questionnaires were used to assess patient and endoscopist satisfaction (Supplementary Tables 1 and 2). Cough score (Table 1), propofol dosage, vital signs, procedure duration, and recovery time were recorded as well. Mean arterial pressure (MAP), HR, and SpO_2_ were recorded at the following time points: at the commencement of the sedation (T1), the moment of procedure starting (T2), 10 min after (T3), 20 min (T4), 30 min (T5), 40 min (T6), 50 min (T7), and the moment of procedure end (T8). The recovery time was calculated as the duration until MAS 9.

On the basis of the pre-experiment data, sample size was calculated. The dose of 1% lidocaine was 4.5 ml/h with an SD of 2.27 ml/h in group E and 5.77 ml/h with an SD of 2.83 ml/h in group P. Assuming a 5% dropout rate, 140 (70 per group) patients were needed with a power of 0.80 and a significance level of 0.05.

In this study, statistical analyses were conducted using IBM SPSS Statistics software (Version 27.0, IBM Corp., Armonk, NY, USA). The Mann–Whitney *U* test was used when data were irregularly distributed, and independent *t*-tests were used for data that were regularly distributed. For nonquantitative data, tables were made and comparisons were evaluated using Chi-square or Fisher exact tests. A *P* value less than 0.05 was considered statistically significant.

## 3. Results

A total of 140 were enrolled and randomly allocated into two groups (group E and group P). Two patients in group E were excluded on account of canceling EBUS-TBNA after ordinary bronchoscopy, and three patients in group P were excluded on account of canceling EBUS-TBNA after ordinary bronchoscopy (*n* = 2) and failing to be followed up (*n* = 1). Finally, 135 patients (group E *n* = 68 and group P *n* = 67) were analyzed (Figure 1).

Participant characteristics and procedure duration of two groups are shown in Table 2. There were no significant differences in age, sex ratio, weight, BMI, ASA classification, smoking and alcoholism habits, and comorbidities. The mean height of patients in group E was 163.2 ± 7.7 cm and in group P was 166.1 ± 7.6 cm (*P*=0.029). The procedure duration had no difference between the two groups (49.0 ± 11.2 min in group E versus 49.7 ± 12.1 min in group P, *P*=0.744).

There were significant differences in the dosage of lidocaine and times of lidocaine sprays between the two groups (Table 3). Patients in group E received less lidocaine (4.36 ml/h (2.67–6.00)) than patients in group P (6.00 ml/h (4.36–7.20), *P* < 0.001). In addition, patients in group E were given less times of sprays (2.18 times/h (1.33–3.00)) than patients in group P (3.00 times/h (2.18–3.60), *P* < 0.001). There was a statistically significant difference in cough score between the two groups (group E 2 (0–4) vs group P 3 (2–4), *P*=0.03). The esketamine dose of group E was 0.52 mg·kg^−1^·h^−1^ (0.49–0.62), and the difference of propofol application between the two groups was statistically significant (3.47 mg·kg^−1^·h^−1^ (3.07–3.97) vs 4.27 mg·kg^−1^·h^−1^ (3.08–4.99), *P* < 0.001). However, there was no difference in endoscopist satisfaction, patient satisfaction, and recovery time between the groups.

Adverse events related to sedation were similar between the two groups. During the procedure, approximately 16.2% of patients in group E and 20.9% of patients in group P experienced hypotension (*P*=0.480). As for esketamine-related psychiatric symptoms, one patients in group E experienced hallucination after the procedure, and there was no statistically difference between the two groups (*P*=1.000).

**Figure 2:**
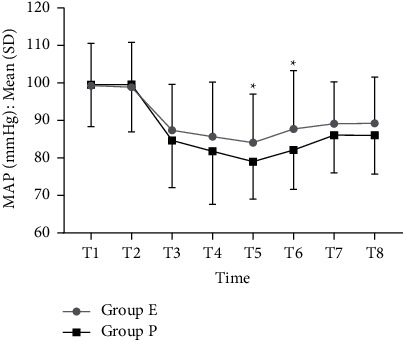
Comparison of MAP between the two groups at different times. MAP, mean arterial pressure. ^*∗*^*P* < 0.05.

Figure 2 showed the MAP of the two groups at different times. The MAP levels at T1 (before sedation), T2, T3, T4, T7, and T8 were comparable in the two groups. Whereas, there was a significant decrease in group P at T5 (79.04 ± 10.01 mmHg in group P versus 84.10 ± 12.91 mmHg in group E, *P*=0.012) and T6 (82.1 ± 10.51 mmHg in group P versus 87.72 ± 15.55 mmHg in group E, *P*=0.026) compared with group E. HR and SpO_2_ did not differ significantly between groups at any time (Supplementary Figures 1 and 2).

## 4. Discussion

In this study, we explored the efficacy and safety of a subclinical dose of esketamine used for deep sedation in the EBUS-TBNA procedure. The results demonstrated that using esketamine in combination can improve the sedative effect, reduce the cough reaction, and maintain more stable blood pressure to some extent. However, we did not observe a reduction in the incidence of sedative-related adverse events.

Endoscopists usually spray local anesthetic through the bronchoscope during bronchoscopy to improve patient tolerance and reduce cough rate [3, 15]. When patients coughed heavily and needed to spray lidocaine during the puncture procedure, the endoscopist had to withdraw the puncture needle first, spray lidocaine, and then insert the puncture needle again and reposition, increasing the procedure duration, number of punctures, and patient expenses. For that reason, the dose and times of lidocaine sprayed by endoscopists through the bronchoscope were conducted as the primary outcome to assess the efficacy of sedation. Based on the results, esketamine application reduced the dosage of lidocaine and times of lidocaine spraying, and decreased cough scores. This outcome was considered to be related to the potent sedative, analgesic, and antitussive properties of esketamine. Currently, there have been no reports of esketamine being used in EBUS-TBNA, but in the case of ketamine, Fruchter et al. discovered that ketamine and fentanyl had similar efficacy and safety for bronchoscopy in an 80-patient trial [16]. Dal et al. reached a similar conclusion comparing ketamine-midazolam with ketamine-propofol during bronchoscopy [17]. We suspected that the effectiveness advantage of esketamine in our study stemmed from its use as an adjuvant rather than an alternative to other sedative or analgesic drugs. The combination of esketamine, propofol, and opioids can maximize sedative and antitussive effects while decreasing drug dosage. However, whether esketamine can truly reduce the number of punctures and increase the accuracy of punctures and the positive rate of diagnosis requires further investigation.

Esketamine has sympathomimetic effects and increases cardiac output in a dose-dependent manner [14], which may counteract the cardiovascular inhibition of propofol. It was observed in our study that although there was no difference in the incidence of hypotension between the two groups, the MAP in group E was higher than that in group P at 30 min and 40 min during the procedure. This is deemed clinically significant and has a potential benefit. The combination of esketamine may have more advantages in maintaining the fragile hemodynamics in frail patients. This hypothesis needs further validation.

Schlatter et al. reported that 42 patients occurred hypoxemia (SpO_2_ < 90%) in their study of 146 adult patients given a propofol-hydrocodone combination for flexible bronchoscopy [18]. In another retrospective study, Müller et al. showed that 72 patients had transient respiratory deterioration and 6 patients needed short time mechanical ventilation under sedation with midazolam, fentanyl, and propofol for flexible bronchoscopy [10]. In our study, both groups had a low incidence of hypoxemia, and no obvious respiratory depression was observed. The main reason for our consideration is that propofol was administered using TCI technology. TCI can more precisely manage the concentration and speed of drug infusion using a computer-controlled drug injection pump, allowing the drug to reach the intended location more safely and rapidly. According to research, the adverse effects of propofol deep sedation have been connected to the infusion mode. Passot et al. found that in direct laryngoscopy with deep sedation, afentanil combined with propofol TCI had less respiratory depression, less body motion, and more stable hemodynamics than manually controlled infusion [19]. Propofol TCI can also be safely used in patients with acute respiratory failure requiring tracheoscopy [20]. The other reason is that we observed that the majority of hypoxemia was caused by airway obstruction in the prone position due to glossoptosis. Treatment of jaw thrust or placement of a nasopharyngeal airway tube can alleviate almost all hypoxemia.

The side effects of esketamine include laryngeal spasms, increased airway secretion, hallucinations, nightmares, and other mental symptoms, which limit its application in adult bronchoscopy sedation. To reduce these adverse effects, all patients were given penehyclidine hydrochloride before the procedure to suppress gland production and a modest dosage of midazolam to prevent esketamine-related mental symptoms. Both oropharyngeal topical anesthesia and transtracheal administration were performed to reduce laryngeal sensitivity. One laryngospasm and one hallucination were found in our study. The patient with laryngospasm had a history of asthma, and the bronchoscope passed through the glottis four times during the procedure, which may be the reason for the laryngospasm. Also, the patient with hallucination was a young male with a large weight, similar to the patient with hallucination observed by Dal et al. [17]. However, we found no evidence that young male patients were more likely to have psychiatric complications injected with esketamine. In our study, there was no statistically significant difference in complications between the two groups, and we considered that small dose esketamine is safe and reliable in EBUS-TBNA.

Our research has limitations as well. First, esketamine can increase the bispectral index (BIS) value for its influence on brain waves [21, 22]; therefore, we cannot use BIS to objectively assess the degree of sedation. As a result, we went with the more subjective MOAA/S. However, the deeper degree of sedation could not be reliably assessed when the score reached 0 (no response after a severe trapezius pressure). We believe that more sophisticated sedation evaluation methods can be addressed in future studies, making the results more comparable. Second, we included patients with ASA II-III and excluded those with severe complications. However, patients above ASA IV are also common in clinical practice. It means that the conclusion cannot be shared among more vulnerable populations.

In conclusion, esketamine combined with propofol TCI deep sedation for EBUS-TBNA can to some extent produce better sedation and more stable blood pressure than propofol TCI alone. The coughing reaction can also be reduced. No reduction in the occurrence of sedation-related side effects was observed.

## Figures and Tables

**Table 1:** Cough score.

Score	0	1	2	3	4
Single cough duration	0 s	<5 s	5−20 s	>20 s	—
Coughing times	0	1-2	3–5	>5	—
Body movement	Not with body movement	Cough action only	Upper limbs movement	Upper and lower limbs movement	Strenuous full body movement

Cough is scored in three aspects: single cough duration, coughing times, and body movement. The final score (0–10) is the sum of the three scores.

**Table 2:** Patient characteristics.

	Group E (*n* = 68)	Group P (*n* = 67)	*P* value
Age (y)	62.9 ± 9.6	65.6 ± 10.2	0.113
Male (*n*, %)	34 (50)	42 (62.7)	0.137
Height (cm)	163.2 ± 7.7	166.1 ± 7.6	0.029^*∗*^
Weight (kg)	61.9 ± 8.9	64.1 ± 8.1	0.148
BMI (kg/m^2^)	23.2 ± 2.7	23.3 ± 2.8	0.945
ASA (*n*, %)			
II	49 (72.1)	49 (73.1)	0.889
III	19 (27.9)	18 (26.9)	
Smoking (*n*, %)	25 (36.8)	35 (52.2)	0.070
Alcoholism (*n*, %)	4 (5.9)	7 (10.4)	0.332
Comorbidities (*n*, %)			
Hypertension	26 (38.2)	23 (34.3)	0.637
Cardiac disease	12 (17.6)	16 (23.9)	0.372
Cerebrovascular disease	7 (10.3)	6 (9.0)	0.792
Diabetes	5 (7.4)	8 (11.9)	0.366
Procedure duration (min)	49.0 ± 11.2	49.7 ± 12.1	0.744

Age, height, weight, BMI, and procedure duration are expressed as mean ± standard deviation. ASA, American Society of Anesthesiologists; BMI, body mass index. ^*∗*^*P* < 0.05.

**Table 3:** Primary and secondary outcomes.

	Group E (*n* = 68)	Group P (*n* = 67)	*P* value
Primary outcome			
Lidocaine dosage (ml/h)	4.36 (2.67–6.00)	6.0 0 (4.36–7.20)	*P* < 0.001^*∗*^
Frequency of spraying (times/h)	2.18 (1.33–3.00)	3.00 (2.18–3.60)	*P* < 0.001^*∗*^
Secondary outcome			
Cough score	2 (0–4)	3 (2–4)	*P*=0.030^*∗*^
Propofol dosage (mg·kg^−1^·h^−1^)	3.47 (3.07–3.97)	4.27 (3.68–4.99)	*P* < 0.001^*∗*^
Endoscopist satisfaction	10 (9–10)	10 (9–10)	*P*=0.264
Patient satisfaction	10 (10–10)	10 (10–10)	*P*=0.303
Recovery time (min)	6 (6–7)	6 (5–7)	*P*=0.116
Sedation-related adverse events (*n*, %)			
Hypoxemia	4 (5.9)	8 (11.9)	*P*=0.243
RR < 7 (times/min)	3 (4.4)	4 (6.0)	*P*=0.718
Hypotension	11 (16.2)	14 (20.9)	*P*=0.480
Hypertension	3 (4.4)	4 (6.0)	*P*=0.718
Tachycardia	2 (2.9)	5 (7.5)	*P*=0.274
Bradycardia	1 (1.5)	0 (0)	*P*=1.000
Malignant arrhythmia	0 (0)	3 (4.5)	*P*=0.119
Laryngospasm	1 (1.5)	0 (0)	*P*=1.000
Hallucination	1 (1.5)	0 (0)	*P*=1.000
Nausea and vomiting	8 (11.8)	5 (7.5)	*P*=0.397

Data are expressed as median (interquartile range) or *n* (%). VAS, visual analogue scale; RR, respiratory rate. ^*∗*^*P* < 0.05.

## Data Availability

The complete data used to support the findings of this study are available from the corresponding author upon request.

## Supplementary Materials

Supplementary Materials Supplementary Table 1: patient satisfaction questionnaire. Supplementary Table 2: endoscopist satisfaction questionnaire. Supplementary Figure 1: comparison of HR between the two groups at different times. HR, heart beat. Supplementary Figure 2: comparison of SpO_2_ between the two groups at different times. SpO_2_, oxygen saturation.
