# Supplementary material for: Esketamine Combined with Propofol TCI versus Propofol TCI for Deep Sedation during Endobronchial Ultrasound-Guided Transbronchial Needle Aspiration: A Prospective, Randomized, and Controlled Trial
Source: Int J Clin Pract. 2023 Dec 11;2023:1155126. doi: 10.1155/2023/1155126 (PMC10728353; doi:10.1155/2023/1155126)
Supplement: Supplementary Materials — Supplementary Table 1: patient satisfaction questionnaire. Supplementary Table 2: endoscopist satisfaction questionnaire. Supplementary Figure 1: comparison of HR between the two groups at different times. HR, heart beat. Supplementary Figure 2: comparison of SpO2 between the two groups at different times. SpO2, oxygen saturation. [file 1155126.f1.zip › Supplementary Table 1-2.docx]

Supplementary Table 1 Patient satisfaction questionnaire

| question | option |
| --- | --- |
| 1. Do you remembe the bronchoscope procedure? | Yes 0  NO 2 |
| 1. Do you have nightmares during the procedure? | Yes 0  NO 2 |
| 1. Do you have hallucination after the procedure? | Yes 0  NO 2 |
| 1. Do you feel nauseous or vomit after the procdure? | none 2  feel nauseous 1  vomit 0 |
| 1. Are you willing to undergo tracheoscope procedure again if needed? | Yes 2  No 0 |

Supplementary Table 2 Endoscopist satisfaction questionnaire

| question | option |
| --- | --- |
| 1. Are you satisfied with the anesthesia procedure? | Very satisfied 3  Satisfied 2  General 1  Not satisfied 0 |
| 1. Are you satisfied with the sedation level? | Very satisfied 3  Satisfied 2  General 1  Not satisfied 0 |
| 1. How many times were you interrupted by coughing or body movement? | 0 time 4  1 time 3  2-3times 2  4-5times 1  More than 6 times 0 |
