# Supplementary figures and images for: Esketamine Combined with Propofol TCI versus Propofol TCI for Deep Sedation during Endobronchial Ultrasound-Guided Transbronchial Needle Aspiration: A Prospective, Randomized, and Controlled Trial
Source: Int J Clin Pract. 2023 Dec 11;2023:1155126. doi: 10.1155/2023/1155126 (PMC10728353; doi:10.1155/2023/1155126)

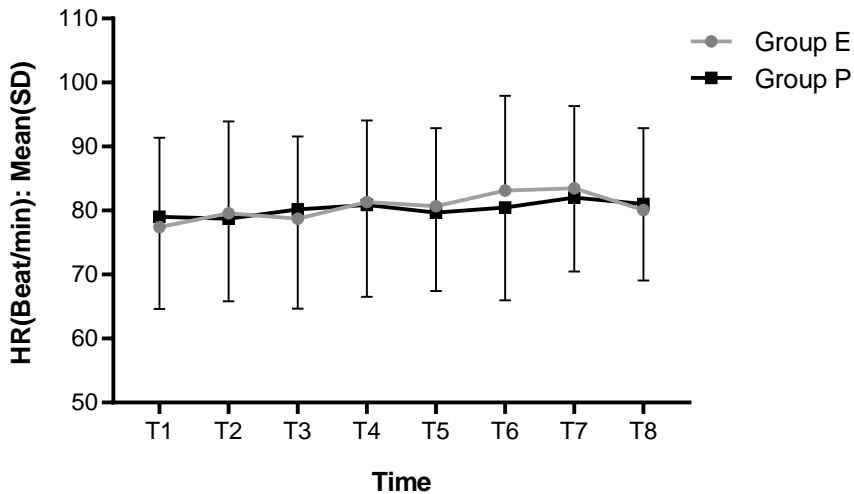

Supplement: Supplementary Materials — Supplementary Table 1: patient satisfaction questionnaire. Supplementary Table 2: endoscopist satisfaction questionnaire. Supplementary Figure 1: comparison of HR between the two groups at different times. HR, heart beat. Supplementary Figure 2: comparison of SpO2 between the two groups at different times. SpO2, oxygen saturation. [file 1155126.f1.zip › Supplementary Figure1.pdf]

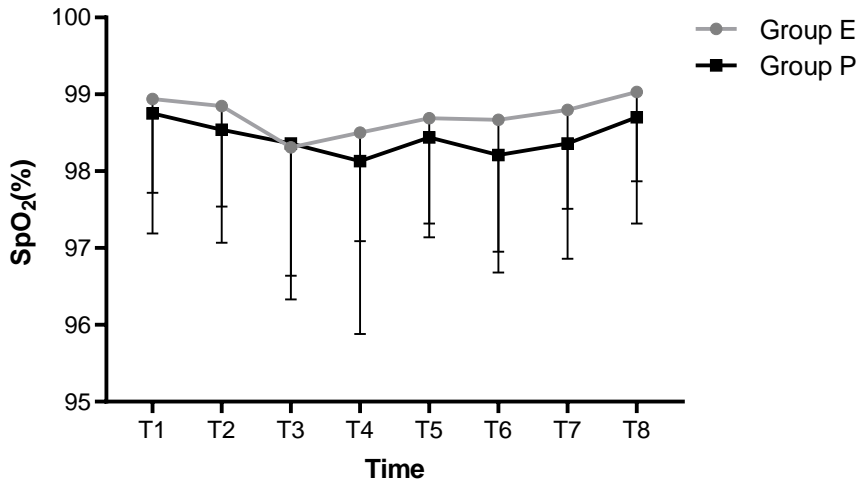

Supplement: Supplementary Materials — Supplementary Table 1: patient satisfaction questionnaire. Supplementary Table 2: endoscopist satisfaction questionnaire. Supplementary Figure 1: comparison of HR between the two groups at different times. HR, heart beat. Supplementary Figure 2: comparison of SpO2 between the two groups at different times. SpO2, oxygen saturation. [file 1155126.f1.zip › Supplementary Figure2.pdf]
